# 2W HBT Power Amplifier Module with Dual Second Harmonic Suppression Technique

**DOI:** 10.3390/s25041231

**Published:** 2025-02-18

**Authors:** Chul-Woo Byeon, Joon-Hyung Kim

**Affiliations:** 1 School of Electronics and Electrical Engineering, College of Engineering, Dankook University, Yongin-si 16890, Republic of Korea; cwbyeon@dankook.ac.kr; 2 Department of Semiconductor Convergence, Chungnam National University, Daejeon 34134, Republic of Korea

**Keywords:** heterojunction bipolar transistor (HBT), linearity, power amplifier (PA), power-added efficiency (PAE)

## Abstract

This paper presents a high-power heterojunction bipolar transistor (HBT) power amplifier (PA) module designed for GSM/EDGE applications. The proposed HBT PA employs a differential output stage that delivers high output power at a low supply voltage. A transformer-based output matching network is employed to combine the differential output signals. Through the selection of an appropriate capacitor value at the transformer’s center tap, linearity is enhanced across a wide bandwidth without requiring additional second harmonic termination. When assembled with a low-pass filter and an antenna switch, the PA module achieves an output power of 36 dBm and a power-added efficiency (PAE) exceeding 40% in GSM mode. In EDGE mode, it delivers an output power of 28.5 dBm with a PAE exceeding 20%. Additionally, the designed PA module achieves an adjacent channel power ratio of −60 dBc at a 400 kHz offset with an output power of 28.5 dBm.

## 1. Introduction

**Figure 1:**
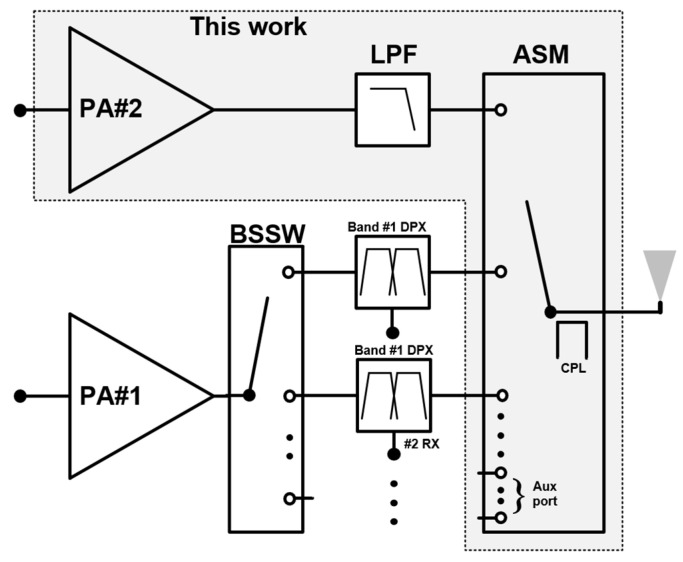
Simplified sub 6 GHz RFFE architecture.

As the demand for higher data rates increases, new radio (NR) technologies, such as 5G, have evolved and now coexist with legacy systems like GSM/EDGE and LTE. This transition necessitates multi-mode and multi-band (MMMB) operation, significantly increasing the complexity of the radio frequency front-end (RFFE) module [1]. For example, a simplified RFFE for low-band (LB) frequencies below 1 GHz is shown in Figure 1. It consists of two power amplifiers (PAs), a band-selection switch (BSSW), a surface acoustic wave (SAW) duplexer (DPX), a low-pass filter (LPF), and an antenna switch module (ASM). To support either LTE or 5G connectivity, the communication signal is amplified by a single LB PA (PA#1) and transmitted through the BSSW, DPX, and ASM. For a legacy service such as GSM/EDGE, a dedicated PA (PA#2) is used due to the high output power requirements. The LPF is necessary to attenuate harmonics generated by the PA output. To accommodate numerous frequency bands and carrier aggregation, a single-pole multiple-throw (SPMT) configuration is essential for the ASM, as shown in Figure 1. However, this increases the insertion loss from the PA output node to the antenna, requiring the PAs to deliver higher output power. For example, assuming an ASM insertion loss of 0.5 dB, designing a GSM/EDGE PA is particularly challenging because it must deliver approximately 36 dBm under a low supply voltage of 3.5 V. This stringent output power requirement poses significant challenges, even when using the GaAs HBT process, which is widely employed for GSM/EDGE PA designs. While enlarging the emitter area of the power stage can increase output power, it also introduces parasitic components due to the larger HBT array. These parasitic components degrade RF performance, preventing a linear increase in output power relative to the incremental emitter area. Furthermore, the enlarged emitter area results in a very low optimum fundamental impedance, which increases the transformation loss in the output matching network. To achieve higher output power with good linearity, a differential power stage based on transformers has recently gained attention [2,3,4,5,6,7]. Compared to a single-ended power stage, the differential power stage can deliver twice the output power at the same size as the individual power stage. This makes them widely used in CMOS PA designs [2,4], and they have been proven effective for HBT PAs in the sub 6 GHz range [5,6,7]. In this paper, we present an HBT PA module capable of delivering a maximum output power of 2W for GSM Class-4 operation, while providing a linear output power of 28.5 dBm for EDGE.

## 2. HBT PA Module Design

**Figure 2:**
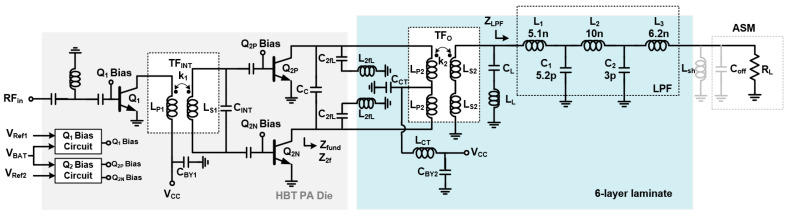
Schematic of the proposed PA module.

Figure 2 illustrates the schematic of the proposed PA module, which consists of an HBT PA die, an LPF, and an ASM. In the HBT PA, while the driver stage is implemented as a single-ended amplifier, the power stage employs a differential pair to achieve a compact die size. The emitter areas are 1440 mm^2^ and 2 × 5850 mm^2^ for the driver stage $Q_{1}$ and the power stage $Q_{2}$, respectively. To meet the stringent requirements for harmonic rejection, the harmonic components in the PA output signal are attenuated by an LPF, which is synthesized using surface-mount device (SMD) components. The ASM is designed as a single-pole ten-throw (SP10T) switch, including forward and reverse coupling ports, and is fabricated using CMOS SOI technology. Each arm of the ASM incorporates a dedicated shunt inductor $L_{sh}$ to cancel out the effect of the off-capacitance $C_{off}$ in the ASM. For simplicity, the equivalent input resistance of the ASM is assumed to be 50 Ω. The measured insertion loss of the ASM is approximately 0.48 dB at 824–915 MHz. The module design, including the HBT PA circuit shown in Figure 2, is characterized using the PathWave Advanced Design System (ADS) and incorporates a finite element method (FEM) simulator for electromagnetic (EM) simulation. The detailed values of the design parameters are summarized in Table 1.

### 2.1. HBT PA Design

**Figure 3:**
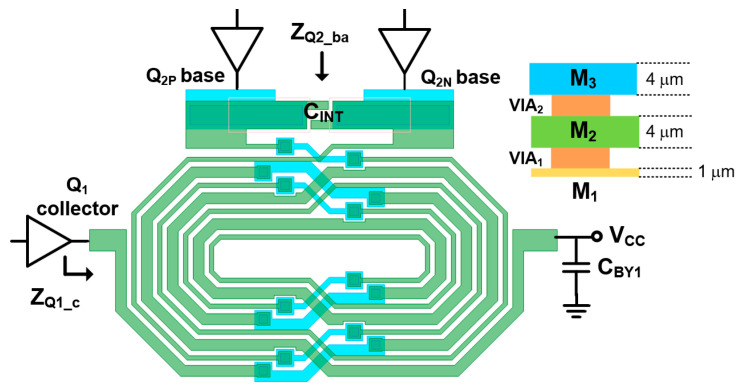
Top view of inter-stage transformer (${TF}_{INT}$).

**Figure 4:**
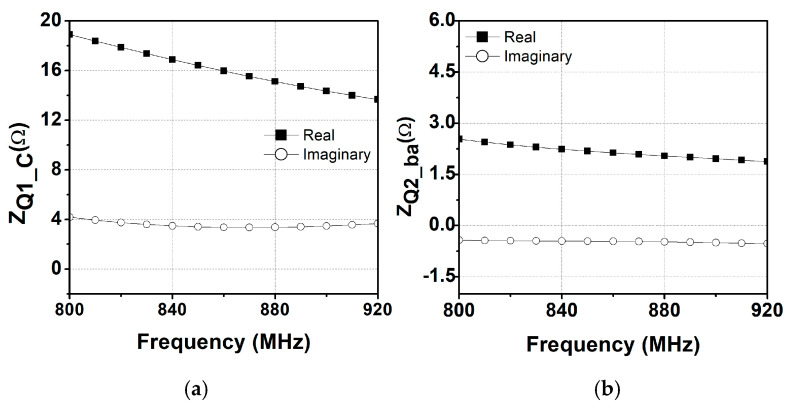
Impedances of $Z_{Q1\_c}$ and $Z_{Q1\_ba}$.

The single-ended output signal from the driver stage is converted into a differential signal using an inter-stage transformer (${TF}_{INT})$ with a supply bypass cap ($C_{BY1})$, as shown in Figure 2. Figure 3 shows the detailed layout of the ${TF}_{INT}$ with the metal stack-up of the HBT process. The HBT process used in this work provides three metal layers: M_2_ and M_3_ are 4 μm thick metals and M_1_ is a 1 μm thin metal. To reduce the parasitic resistance of the transformer, stacked M_1_ and M_2_ layers are connected through vias, while M_3_ is used as a bridge metal for routing. As a result, the simulated inductance of the primary coil $L_{P1}$ and secondary coil $L_{S1}$ is 2.5 nH and 3.1 nH, respectively, leading to a high coupling factor $k_{1}$ of 0.75 at 900 MHz. For these inductances, the parasitic resistances are only 2.2 Ω and 3.9 Ω, respectively. Figure 4a shows the simulated impedance observed from the collector of the driver stage $Z_{Q1\_c}$, while Figure 4b shows the impedance observed from the base node of the power stage $Z_{Q2\_ba}$. While the real part of $Z_{Q1\_c}$ is observed from 14 Ω to 18 Ω, the imaginary part remains approximately 4 Ω across the given frequency band, ensuring broadband operation. Through the addition of a $C_{INT}$ of 2 pF, the imaginary part of $Z_{Q2\_ba}$ is further flattened to approximately 0.5 Ω, while the real part of $Z_{Q1\_ba}$ is approximately 2 Ω, which closely matches the optimum source impedance of the power stage.

### 2.2. Second Harmonic-Tuned Output Matching

**Figure 5:**
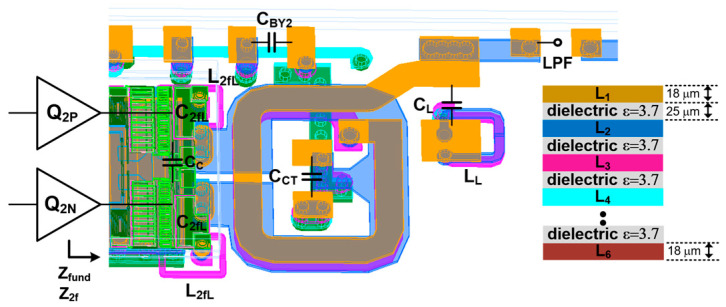
Top view of proposed output matching.

**Figure 6:**
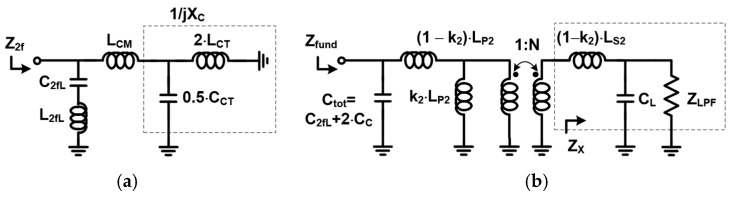
Simplified (**a**) half-circuit for even-order frequency and (**b**) half-circuit for fundamental frequency.

To meet the specification of harmonic power less than −40 dBm, a conventional fifth-order LC LPF is used, as shown in Figure 2. The simulated input impedance of the LPF $Z_{LPF}$ is 22−j0.1 Ω, with the component values indicated in Figure 2. Under these conditions, the simulated attenuation exceeds 20 dB at both the second and third harmonic frequencies, while maintaining a low insertion loss of 0.67 dB. The output transformer ${TF}_{o}$ then converts $Z_{LPF}$ into the optimum fundamental impedance $Z_{fund}$, which is simulated to be as low as 2 Ω to deliver the maximum output power of 36 dBm, accounting for the insertion losses of both the LPF and ASM. Figure 5 shows the layout of ${TF}_{o}$ along with the metal stack-up of the laminate. A six-layer lamination process is utilized for PA module implementation, where three metals are dedicated to the construction of ${TF}_{o}$. The thickness of the copper is 18 mm. While the $L_{1}$ and $L_{3}$ copper layers are used for the secondary coil, $L_{2}$ is used for the primary coil. The width of the primary metal is set to 200 µm for the handling of a high DC current, while the secondary one is set to 140 µm, which is much narrower, to avoid the misalignment effect of the lamination process. Such a structure enhances the broadside magnetic coupling between the primary and secondary coil, resulting in a high coupling factor $k_{2}$ = 0.82. An inductance of $L_{P2}$ of 0.3 nH and that of $L_{S2}$ of 1.1 nH are obtained, respectively. Since a second HT is widely adopted in PA design to improve linearity [5,7,8,9], a series resonator composed of a capacitor and an inductor is utilized to reduce the second harmonic impedance, as shown in Figure 5. To ensure a compact module size, the capacitor $C_{2fL}$ is implemented using an MIM capacitor within the HBT die, while the inductor $L_{2fL}$ is realized using a thick metal layer on the laminate. This harmonic trap offers low loss due to its high quality factor. However, it exhibits a narrower bandwidth, which is insufficient to eliminate the second harmonic component across the entire frequency band. To suppress the second harmonic component corresponding to the fundamental frequency within the 824 MHz–915 MHz range, the implementation of an additional LC second HT could be considered. However, such an addition increases both the insertion loss and the complexity of the module. To address this, a novel approach is proposed to generate an additional pole at the second harmonic frequency. This involves utilizing the even-mode characteristic of the output transformer ${TF}_{o}$ combined with an appropriately chosen center-tap capacitor $C_{CT}$, as illustrated in Figure 5. Assuming that the capacitance of $C_{BY2}$, a supply bypass capacitor, is very large, the simplified equivalent half-circuit for even-order frequencies is modeled as shown in Figure 6a. The first resonance pole at the lower second harmonic frequency is generated by the series combination of $C_{2f,L}$ and $L_{2f,L}$. An additional resonance pole at the higher second harmonic frequency is formed by $L_{CM}$, $L_{CT}$, and $C_{CT}$, where $L_{CM}$ is a common-mode inductance of ${TF}_{o}$ and $L_{CT}$ is an inductance of a metal trace between the center tap of ${TF}_{o}$ and $C_{BY2}$. Assuming that $X_{C}$ is an admittance of parallel $L_{CT}$ and $C_{CT}$ as shown in Figure 6a, the required value of $C_{CT}$ to resonate at an additional second harmonic frequency of ${2f}_{H}$ is given as

(1) $$C_{CT}=\frac{1}{\omega^{2}}\left(\frac{2}{L_{CM}}+\frac{1}{L_{CT}}\right)$$

**Figure 7:**
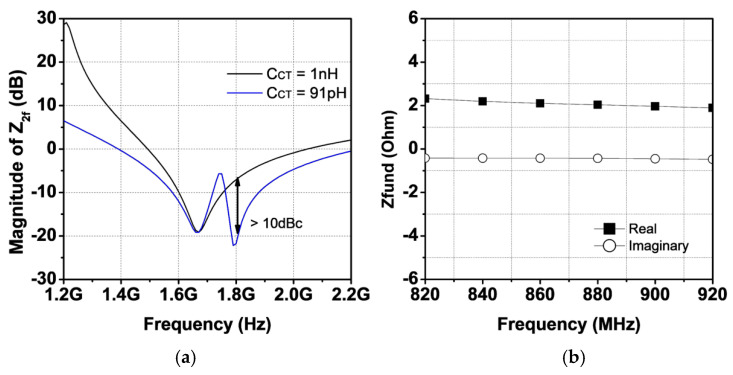
Simulated (**a**) magnitude of $Z_{2f}$ and (**b**) $Z_{fund}$.

**Figure 8:**
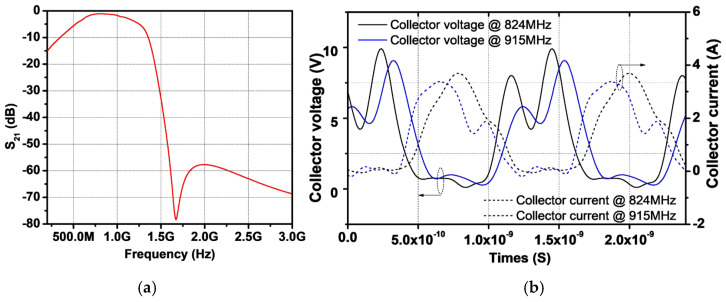
(**a**) Frequency response of output matching (**b**) voltage and current waveform at collector of power stage.

**Figure 9:**
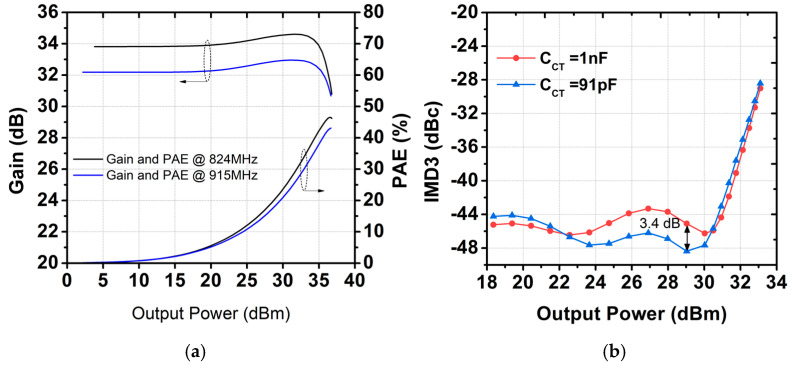
(**a**) Gain and PAE as function of output power and (**b**) IMD3 performance as function of output power.

With an $L_{CT}$ of 0.2 nH and $C_{CT}$ of 91 pF, the magnitude of $Z_{2f}$ is shown in Figure 7a. Compared with the magnitude $Z_{2f}$ with a $C_{CT}$ of 1 nF, it is observed that an additional pole at 1.8 GHz is generated with a $C_{CT}$ of 91 pF, reducing the magnitude of $Z_{2f}$ by as much as 10 dB. For the fundamental load impedance analysis, $L_{CT}$ and $L_{L}$, assumed to be small inductance values, are ignored. Here, $L_{L}$ represents the inductance connected with the load capacitor $C_{L}$ for the third harmonic trap. The equivalent half-circuit at the fundamental frequency is shown in Figure 6b. The fundamental load impedance is given as

(2) $$Z_{fund}\approx (\frac{Z_{x}}{N^{2}}+(1-k_{2})L_{p2})//j\omega C_{tot}$$

where $Z_{x}$ is the impedance seen from secondary coil toward the load and $C_{tot}$ represents the value of $C_{2fL}+C_{C}$, as shown in Figure 6b. With a $C_{L}$ of 8.8 pF, the simulated fundamental load impedance seen from the collector of the power stage, $Z_{fund}$, is shown in Figure 7b. The simulation reveals that the real part of $Z_{fund}$ is close to 2 Ω as targeted, whereas the imaginary part of $Z_{fund}$ is close to zero across the operating frequency. Figure 8a shows the frequency response of the proposed output network. A simulated insertion loss of 1.13 dB is achieved, with attenuation at the second and third harmonic frequencies as low as 60 dB. To verify proper operation for a second harmonic short, the voltage and current waveforms are shown in Figure 8b. As designed, the voltage waveform is squared for both frequencies, while the current waveform forms a half-sinusoidal shape. Figure 9a presents the simulated gain and PAE as functions of output power. The proposed PA module delivers a saturated output power of 36.5 dBm for both frequency bands. The peak PAE is 46% at 824 MHz and 43% at 915 MHz. Additionally, Figure 8 shows a comparison of third-order intermodulation distortion (IMD3). Using a 1 MHz tone spacing at 900 MHz, the proposed dual harmonic trap improves IMD3 by 3.4 dB at an output power of 29 dBm compared to a single harmonic trap.

### 2.3. Implementation of HBT PA Module

**Figure 10:**
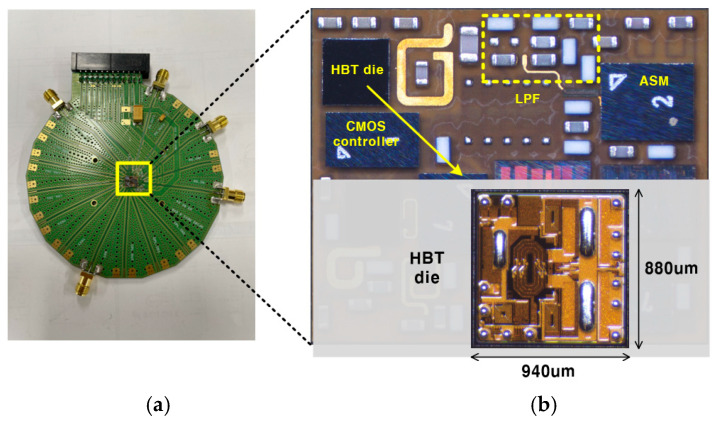
Photograph of (**a**) evaluation board and (**b**) PA module including chip photograph of HBT PA die.

Figure 10a shows a photograph of the evaluation board (EVB) used for testing the PA module, as depicted in Figure 6b. The EVB is constructed using FR-4 material with four metal layers. The top metal layer is utilized for RF signal routing, while the inner layers serve as the circuit ground, DC supply line, and control signal pathways. The proposed HBT PA was fabricated using a commercial 2 mm GaAs HBT technology with Cu bump pillars. The PA die occupies an active area of 0.94 × 0.88 mm^2^, as shown in Figure 10b. Compared with a bond-wire design, Cu bump pillars not only maintain a compact form factor but also enhance thermal management performance. To further improve thermal conductivity, large bar-type Cu bump pillars are directly placed on the emitter of both the driver and power stages, as illustrated in Figure 10b. The bias circuit’s reference voltage is regulated and supplied by a CMOS controller fabricated using bulk CMOS technology. Additionally, the ASM is fabricated in CMOS SOI technology to achieve low insertion loss. The LPF is implemented with 0402 high-Q inductors and high-Q capacitors. All chips are attached to the laminate using a flip-chip bump process.

## 3. Measured Results

**Figure 11:**
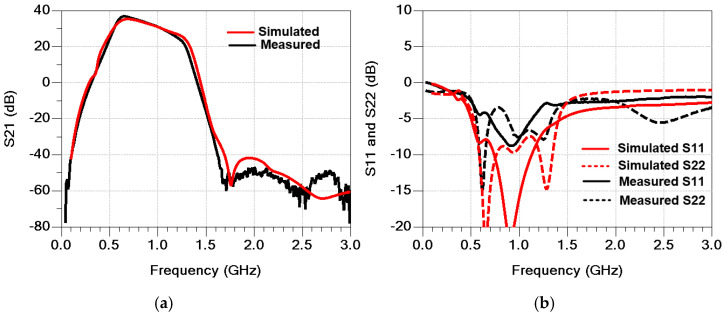
Measured s-parameter results compared with simulation results (**a**) S21 and (**b**) S11 and S22.

**Figure 12:**
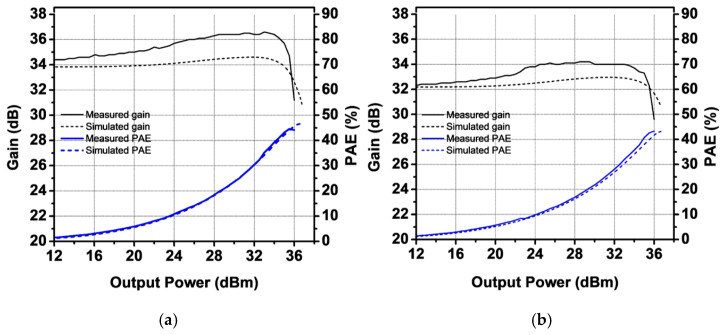
Measured gain and PAE as function of output power (**a**) at 824 MHz and (**b**) at 915 MHz.

**Figure 13:**
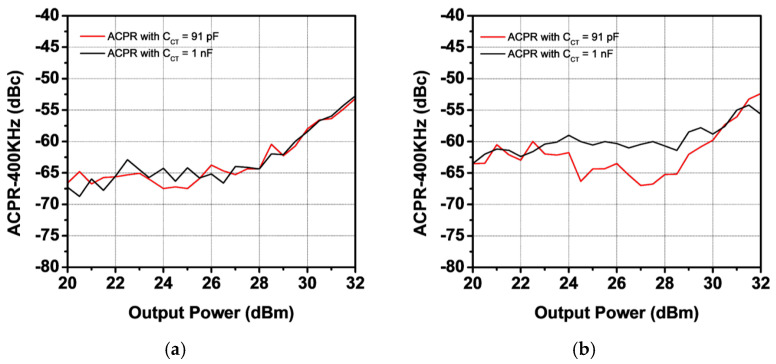
Measured ACLR-400 kHz (**a**) at 824 MHz and (**b**) at 915 MHz.

Figure 11 shows the measured S-parameters of the PA module compared with the simulation results. The quiescent current was set to 250 mA, with VCC at 3.5 V and VBAT at 3.8 V. The measured S21 values are 34.4 dB and 32.7 dB at 824 MHz and 915 MHz, respectively. The harmonic rejection at both the second and third harmonic frequencies is less than −80 dBc. The measured S11 is lower than −8 dB, while S22 is below −4 dB. The measured S11 and S22 are slightly different from the simulation results because the simulation was performed taking into consideration only the loss of the in/out metal lanes in the EVB board. The large-signal performance was characterized for two modes: GSM mode and EDGE mode. Figure 12 presents the measured gain and PAE as functions of output power using a GMSK signal. At an output power of 36 dBm, the measured power gain is 31.2 dB and 29.6 dB for 824 MHz and 915 MHz, respectively, with corresponding PAE values of 44.6% and 43.3%. At the saturated output power, the second and third harmonic power levels are below −50 dBm and −43 dBm for both frequency bands. The received band noise at a 20 MHz offset frequency is as low as −88 dBm/100 kHz. Figure 13 shows the measured ACLR at a 400 kHz offset frequency (ACLR-400 kHz) as a function of output power. The ACLR-400 kHz was measured with two different values of $C_{CT}$ to verify improvement in the second harmonic termination effect. While the PA module with different $C_{CT}$ values provides similar ACLR-400 kHz performance at 824 MHz, the ACLR-400 kHz of the PA module with a $C_{CT}$ of 91 pF at 915 MHz improves by as much as 4.8 dBc compared with that of the PA module with a $C_{CT}$ of 1 nF, as shown in Figure 13a. The large-signal performances of the proposed PA module are summarized in Table 2, compared with previous work, including industry products.

## 4. Conclusions

In this paper, the design of a 2 W high-power HBT PA module is presented. A differential scheme for a power stage was adopted to deliver high power under a low supply voltage condition. For compact implementation, single-to-differential matching was realized using an on-chip transformer. The differential output signal of a power stage is combined by an output transformer with a properly configured center-tap capacitor. This configuration provides dual second harmonic termination for different operating frequency bands, resulting in improved linearity for the EDGE signal. The proposed PA was fabricated using commercial HBT technology and assembled with a CMOS controller, LPF, and SOI switch. The PA module delivered a peak output power of 36 dBm with a PAE exceeding 40% in GSM mode. In EDGE mode, linearity was improved by 4.8 dBc at an output power of 28.5 dBm. At this output power, for both modes, all other system specifications were satisfied.

## Figures and Tables

**Table 1:** Parameters of designed components.

Parameters	Value	Parameters	Value
$L_{P1}$	3.3 nH	$k_{2}$	0.82
$L_{S1}$	2.6 nH	$C_{CT}$	91 pF
$k_{1}$	0.75	$L_{CT}$	0.4 nH
$L_{P2}$	0.85 nH	$C_{L}$	8.8 pF
$L_{S2}$	2.9 nH	$L_{L}$	0.35 nH

**Table 2:** Performance comparison among 2G GSM/EDGE PAs.

Ref.		[3]	[4]	[10]	[11]	[12]	This work	
Process		180 nm CMOS	153 nm CMOS	GaAs HBT	GaAs HBT	GaAs HBT	GaAs HBT	
Harmonic filter		No	Yes	No	No	Yes	Yes	
Antenna switch		No	Yes	No	No	Yes	Yes	
Frequency (MHz)		800/850	850	814–915	824–915	824–915	824–915	
VDD (V)		3.5	3.5	3.5	3.5	3.5	3.5	
Reference output		PA output	Mod. output	PA output	PA output	PA output	PA output ^1^	Mod. output
GMSK GSM /EGSM	Psat (dBm)	34.5	34	34.3	35	35	37.15	36
	PAE (%)	55	36.8	57.5–62.2	55	55	54.5–57	41.8–43.7
	Harmonic, 2fo (dBm)	-	<−30	-	−10	−10	-	<−50
	Harmonic, 3fo (dBm)	-	<−30	-	−15	−15	-	<−43
	Rx band noise (dBm/100 kHz) @20 MHz offset	−86	−84	-	−87	−87	-	−88
EDGE GSM /EGSM	Pout (dBm)	28.5	28	29	29	29	29.65	28.5
	PAE (%)	22	-	33–35	28	28	27.3	21
	ACPR (dBc) @400 kHz	−57	−61	−52.8	-	−60	-	<−61
	ACPR (dBc) @600 kHz	−75	-	-	-	−70	-	<−70

^1^ Calculated PA output with an ASM loss of 0.48dB and LPF loss of 0.67 dB. Abbreviations: Psat—saturated output power; Mod.—module; ACPR—adjacent channel power ratio; Rx—receiver.

## Data Availability

Data are contained within this article.
